# How Neighborhood Effects Vary: Childbearing and Fathering among Latino and African American Adolescents

**DOI:** 10.3390/healthcare6010007

**Published:** 2018-01-18

**Authors:** Jessica L. Lucero, Anna Maria Santiago, George C. Galster

**Affiliations:** 1 Department of Sociology, Social Work, and Anthropology, Utah State University, Logan, UT 84322, USA; 2 School of Social Work, Michigan State University, East Lansing, MI 48824, USA; santia63@msu.edu; 3 Department of Urban Studies and Planning, Wayne State University, Detroit, MI 48202, USA; george.galster@wayne.edu

**Keywords:** adolescent childbearing, adolescent fathers, African Americans, emerging adulthood, Latinos, neighborhood effects

## Abstract

This study examines what neighborhood conditions experienced at age 15 and after are associated with teen childbearing and fathering among Latino and African American youth and whether these neighborhood effects vary by gender and/or ethnicity. Administrative and survey data from a natural experiment are used for a sample of 517 Latino and African American youth whose families were quasi-randomly assigned to public housing operated by the Denver (CO) Housing Authority (DHA). Characteristics of the neighborhood initially assigned by DHA to wait list applicants are utilized as identifying instruments for the neighborhood contexts experienced during adolescence. Cox Proportional Hazards (PH) models reveal that neighborhoods having higher percentages of foreign-born residents but lower levels of social capital robustly predict reduced odds of teen parenting though the magnitude of these effects was contingent on gender and ethnicity. Specifically, the presence of foreign-born neighbors on the risk of teen parenting produced a stronger dampening effect for African American youth when compared to Latino youth. Additionally, the effects of social capital on teen parenting were stronger for males than females.

## 1. Introduction

For nearly three decades, scholars in the United States and Western Europe have highlighted the connection between early parenting and growing up in impoverished neighborhoods [1,2,3]. Spurred by Wilson’s [4] call to consider the influence of spatially concentrated disadvantage on family formation as well as public and political outcry over record high teen childbearing rates in the 1990s, a number of neighborhood effect studies on teen parenting emerged [1,2,5] Despite a promising downward trend in teen childbearing rates since the 1990s, substantial racial and ethnic disparities remain in the U.S.—these disparities raise questions about the intersection of class and race at both the individual and neighborhood level [6]. 

The neighborhood effects literature links neighborhood structural characteristics, such as concentrated poverty or affluence, economic opportunities and labor force participation, residential instability, and racial/ethnic heterogeneity, to increases in teen risky sexual behavior, pregnancy, and childbearing [3,5,7]. However, few studies have examined the neighborhood contexts associated with teen fathering, and even fewer have simultaneously examined multiple neighborhood conditions or controlled for the bias introduced from unobserved parental characteristics that may be correlated both with residential location and teen parenting [8]. Using an ecological systems framework and drawing on collective socialization and social control theories, this study investigates the neighborhood contexts associated with teen childbearing and fathering (occurring between the ages of 15 and 19) among low-income, Latino and African American youth. The purpose of this study is twofold: (1) To obtain an unbiased measure of the causal influence of a variety of neighborhood indicators on teen parenting; and (2) to determine if these effects vary by ethnicity and gender. Our study is distinguished by its use of data from the *Denver Child Study* [9], a mixed methods retrospective study using a natural experiment in Denver, CO, in which low-income families and their children were quasi-randomly assigned to public housing in an array of socioeconomically and ethnically diverse neighborhoods.

## 2. Literature Review

### 2.1. Theoretical Frameworks for Understanding How Neighborhoods Might Affect Teen Parenting

According to ecological systems theory, an adolescent’s developmental outcomes are shaped through complex, and mutually reinforcing proximal (e.g., family) and distal (e.g., neighborhood) forces [10]. Recent neighborhood theorists have delineated the potential causal mechanisms that relate neighborhood context with individual adolescent outcomes [11,12,13]. The neighborhood effect mechanisms most often cited and theoretically relevant for teen parenting are collective socialization and social control. 

### 2.2. Collective Socialization and Social Control

Theories of collective socialization have long been used to frame neighborhood effect studies on teen fertility outcomes [4,5,14,15]. The attitudes, values, behaviors and expectations that youth hold about teen childbearing and fathering are shaped in large measure by three dimensions of collective socialization: role-modeling, monitoring, and cohesion [16]. Diminished economic opportunity in disadvantaged neighborhoods may trigger support for early parenthood. When these opportunities are still present, neighborhood adults can model different pathways to adulthood, such as continuing education and work. In the absence of viable opportunities for youth, however, adult role models in the community may reinforce local norms that condone early and/or unmarried parenting [4]. In turn, this lack of opportunities decreases community social capital, diminishes the effectiveness of local institutions, and weakens informal social support systems that may otherwise keep adolescents from expeditiously transitioning to adulthood through parenthood [17]. For example, a number of earlier studies note the inverse association between teen childbearing and neighborhood affluence, suggesting the mechanism of collective socialization [1,2,18]. In more affluent neighborhoods, where most neighborhood adults are steadily employed, teens are more likely to adhere to the social norms that are modeled by adults and engage in behaviors that support their success in school and diminish their risk for early parenting [4,19]. 

In structurally disadvantaged neighborhoods (e.g., those characterized by high poverty and high residential turnover) there are often weaker social ties, fewer external resources, and diminished capacity for enforcing shared norms and values that protect neighborhood residents from deleterious outcomes [4,20,21]. The presence of adults in the neighborhood who collectively monitor adolescents can serve as an important form of social control—and neighborhood social control is strengthened when social cohesion is also present. In other words, neighborhoods that are characterized by trust and cohesion are more effective at social control strategies such as establishing pro-social norms through adult modeling (e.g., discouraging teen childbearing by modeling alternative pathways to adulthood). It is important to note how neighborhood ethnic and immigrant composition may operate under a collective socialization framework. Social cohesion may be stronger in immigrant communities with shared cultural values and stronger ties that accompany co-ethnicity [22]. Zhou [23], suggests that immigrant communities have the capacity to mobilize resources, monitor and enforce pro-social values, and scaffold children’s development in a manner that protects children despite the risks that structural neighborhood disadvantage may present. 

In short, structural characteristics of neighborhoods may influence key social dimensions in a neighborhood that are associated with teens’ risk for early parenting. Here we focus on collective socialization theories that suggest that occupational prestige, social capital, ethnic/racial and immigrant composition, and concentrated disadvantage influence a neighborhood’s capacity for protecting or placing youth at risk of early parenting. 

### 2.3. Prior Evidence for Neighborhood Effects on Teen Parenting

#### 2.3.1. Empirical Evidence

Many previous studies on neighborhood effects and teen childbearing used national datasets, such as the Panel Study on Income Dynamics (PSID) and attached Census-derived indicators of neighborhood conditions (e.g., [18,24]). These studies found neighborhood poverty or some combination of indicators representing concentrated disadvantage (e.g., female headship, unemployment, residential instability, welfare receipt) consistently increased the risk of teen childbearing [24,25,26,27,28,29]. Recent evidence from Wodtke [3], suggests that sustained exposure to neighborhood poverty places adolescents at even greater risk of teen childbearing.

#### 2.3.2. Variation in Neighborhood Effects

Recently, the health literature has underscored the necessity of considering differential exposure and differential effects across groups. In other words, racial/ethnic groups may be exposed to different opportunity structures and risks related to neighborhood, and racial/ethnic groups may be exposed to similar risks and still experience differential effects [30]. In light of this perspective, we might expect there may be important variations in neighborhood effects based on race and gender. 

Compared to teen childbearing, relatively few studies have documented the neighborhood contexts associated with teen fathering [8,14]. Results from these studies showed that levels of neighborhood unemployment, structural disadvantage, and neighborhood violence were positively associated with teen fathering. Although neighborhood effects studies that explicitly focus on teen childbearing and fathering have relatively similar findings, Sharkey and Faber [31], highlight the importance of considering gender differences in both the magnitude of neighborhood effects and their underlying mechanisms. Research suggests collective socialization processes may look different for girls and boys. For example, living in neighborhoods characterized by affluence may increase young women’s feelings of safety and decrease the pressure they feel to engage in early sexual activity whereas the same environment may increase young men’s feelings of marginalization and the need to prove their manhood via fatherhood [32,33]. Others have found similar gendered differences in neighborhood effects whereby perceptions of neighborhood safety influence later physical health outcomes for females [34].

Beyond gender, there is a lack of consensus regarding how neighborhood effects on teen childbearing and fathering differ by ethnicity. Several studies suggest that concentrated disadvantage increases African American and Latino teens’ likelihood of bearing or fathering a child by constricting their opportunity structures and limiting other viable paths to adulthood [6]. (see Penman-Aguilar et al., for review). However, Driscoll, Sugland, Manlove, and Papillo [35], note that neighborhoods with better opportunity structures protected Latinas from teen childbearing but not African American girls. Additionally, Kirby, Coyle, and Gould [36], found that higher levels of Latino male employment were associated with higher rates of teen childbearing by Latinas. There appears to be similar in the literature by ethnicity—for African American youths, teen parenting is most often linked to limited opportunities and subsequent expedited transitions to adulthood. Although few studies include Latino youths, some report similar findings to African American samples [37], while others [35,36], highlight important differences. On the whole, there appears to be enough mixed findings regarding ethnicity and neighborhood effects on teen parenting risk to warrant further research. 

#### 2.3.3. Methodological Considerations

According to prominent reviews, there are numerous methodological barriers that impede accurate estimates of neighborhood effects [12,38,39,40]. In particular, geographic selection bias has remained an empirical challenge often left unaddressed in the literature, including studies examining neighborhood effects on teen childbearing and fathering. Geographic selection bias refers to the self-selection of individuals into neighborhoods based on unmeasured personal characteristics or motivations that also affect the outcome being investigated [41]. As a result, the independent causal effect of neighborhood cannot be accurately estimated because of the lack of adequate control variables. 

To date, only a handful of studies on teen childbearing and fathering have empirically addressed the issue of geographic selection bias [8,24,42,43,44]. These studies show mixed results. For example, Plotnick and Hoffman [43], reported that independent neighborhood effects disappeared when using a fixed effects model while Harding [24], found that neighborhood effects remained significant despite adjusting for selection bias using a counterfactual framework and propensity score matching.

There is increasing consensus that experimental designs characterized by random or quasi-random assignment to neighborhoods produce the most unbiased estimates of neighborhood effects [11,45]. However, the only example of this design in the wider neighborhood effects literature is the Moving to Opportunity (MTO) demonstration [44]. The MTO demonstration randomly assigned residents living in distressed public housing to one of three groups: (1) control; (2) rental voucher; and (3) rental voucher plus relocation counseling with the condition of moving to a low-poverty (below 10%) neighborhood and remaining there for at least one year. Results showed that girls in the experimental group whose parents moved to low-poverty neighborhoods felt safer and less pressured to engage in early sexual activity (and thus early pregnancy and childbearing) in their new neighborhoods [46]. However, these findings have been complicated by the substantial residential mobility occurring among study participants, modest differences among groups in many neighborhood indicators, and an inability to “unbundle” aspects of environmental context [47]. In other words, MTO left many questions unanswered regarding the causal mechanisms underlying purported neighborhood effects. 

### 2.4. Present Study

Taken together, the literature on the effect of neighborhood environments on teen childbearing and fathering leaves many lingering questions. The aforementioned studies have provided suggestive results of associations between census tract indicators and teen childbearing and fathering rates. How much of these correlations are due to causation instead of geographic selection bias remains unclear, however, given the contradictory findings discussed here. Very few studies have examined teen fathering, and only one employed a method yielding plausibly causal effects [8]. Finally, potential differences in neighborhood effects on teen parenting for Latino and African American teens have not yet been probed. 

Our study addresses these aforementioned gaps in the extant literature. It addresses the challenge of geographic selection bias by utilizing a natural experiment that mimics random assignment. We employ instrumental variables with exogenous contextual variation identified by using characteristics of the neighborhoods first offered by the Denver Housing Authority to eligible households. Specifically, in response to these gaps, our study attempts to answer the following research questions: (1) What neighborhood conditions experienced at age 15 and after are associated with teen childbearing and fathering among Latino and African American youth? and (2) Do these neighborhood effects vary by gender and/or ethnicity?

## 3. Methods

### 3.1. The Natural Experiment in Denver

The *Denver Child Study* derives its study population from a natural experiment involving the Denver (CO) Housing Authority’s (DHA) conventional and dispersed housing programs [9]. Beginning in 1969, the DHA initiated a dispersed housing program that provides scattered-site, single-family and small-scale, multi-family housing opportunities to 1500 low-income households—this is in addition to approximately 3000 units of conventional public housing. As a result, subsidized housing units are found in 60% of all neighborhoods that are part of the congruent City and County of Denver. From 1987 onwards, public housing applicants who rose to the top of a common residential wait list were offered vacant DHA-managed housing units in either conventional or dispersed programs that met their requirements based on family size and gender of children. Wait list applicants who refused their initial offer received a second offer for the next appropriate available unit. Applicants who refused this second offer returned to the bottom of the wait list, creating an additional wait for housing placement of a year or more. Although 75% of all DHA applicants accepted housing units in their initially assigned neighborhoods, applicants who did not prompted us to assess the DHA initial assignment process more closely.

We conducted an array of statistical balancing tests to ascertain the extent to which applicants were quasi-randomly assigned to DHA housing units; these tests and results are described in detail by Galster and Santiago elsewhere [48]. Findings suggest that, with one notable exception related to African American ethnicity, initial assignment from the wait list to a DHA housing unit mirrored random assignment of households to Denver neighborhoods. African American households tended to be assigned to Denver neighborhoods with higher percentages of African Americans. To address this potential for selection bias, we control for African American ethnicity in our statistical analyses, either with dummy variables in the full sample model or via stratification by ethnicity. 

### 3.2. Denver Child Study Retrospective Survey

The primary source of data for the *Denver Child Study* was a retrospective survey administered to current and former DHA residents identified as parents/caregivers (“caregivers” hereafter) whose families entered DHA during the period between 1 January 1987 and 31 December 2005; had resided in DHA for a minimum of two years; had at least one child under the age of 18 when they moved into DHA; and identified as Latino or African American [9]. Surveys were administered over the phone or in-person during the period between April 2006 and February 2008 [9]. A total of 706 caregivers and their 1706 children aged 2 through 18 met all of the eligibility criteria for the larger Denver Child Study, yielding a final response rate of 57%. In our assessment of respondents vs. non-respondents, we found that both groups had statistically similar incomes and family characteristics. However, survey respondents had longer average tenures in DHA housing; were more likely to be female, single heads of households, and were more likely to be African American when compared with survey non-respondents. 

Caregivers were asked about their children’s health, education, violence exposure, risky behaviors, employment during adolescence and young adulthood, and marriage and childbearing. Additionally, residential histories from birth through age 18 or the child’s age of time of the survey (if the child was younger than 18) were compiled for all eligible children in the household. Finally, respondents were asked to provide extensive details about all of the neighborhoods in which the children lived during childhood as well as detailed information about the characteristics of all members of the household corresponding to each place of residence. These data were compiled into a database which contained information about child, caregiver, household and neighborhood characteristics for each year of a child’s life (for more details see [9]).

Since the outcome for this study is teen childbearing/fathering (occurring between the ages of 15 and 19), the analysis sample from the *Denver Child Study* used in this study includes only youth who were: (1) Aged 15 or older at the time of the retrospective survey; (2) were randomly assigned to a DHA neighborhood prior to becoming a teen parent; (3) resided in DHA housing for a minimum of 2 years; and (4) had complete information for all variables used in the analysis. These criteria produced a final analysis sample of 517. Given the retrospective nature of the study as well as the years for which eligible participants could have lived in DHA, it is important to understand that youths in our analysis sample were not all aged 15 to 19 at one literal point in time, but rather, were at least age 15 or older at the time of the survey. Theoretically, a caregiver could have moved into DHA in 1995 with their then 15-year-old child. This same child would have been age 26 at the time of the survey and their caregiver would have reported retrospective residential history information for that child from1995. 

The dependent variable, teen childbearing/fathering, was derived from the following survey question posed to caregivers for children aged 15 or older at the time of survey: “Have any of your CHILDREN given birth to or fathered children of their own?” If caregivers answered affirmatively, they were asked at what age their child first birthed or fathered a child. These data enabled us to identify whether youth became a teen parent and when this occurred during adolescence. Those who gave birth or fathered a child between age 13 and 19 were identified as teen parents.

### 3.3. Youth, Caregiver and Household Measures

Our analytical models control for an array of youth, caregiver and household characteristics. We controlled for the youth’s gender and ethnicity using three dummy variables for Latina female, Latino male, and African American female; the reference category was African American male. We included an indicator of the child’s age at time of initial assignment in DHA in order to ascertain any associations between the when children were first exposed to the conditions present in the randomly assigned neighborhood setting. The analytical models also control for caregiver age when the child was born, caregiver educational attainment operationalized using a dummy variable reflecting completion of a high school diploma or higher by the time the child was age 15, and average caregiver earnings during high school. 

### 3.4. Neighborhood Measures

All residential history information obtained from caregivers was verified and then geocoded using the U.S. Bureau of the Census’ American FactFinder website utility [9]. Approximately 92% of all residential locations identified by caregivers were matched to a census tract that then permitted us to link these locations to a rich set of census and non-census neighborhood indicators. Because some youth moved to neighborhoods that did not exist prior to 2000, we could not geocode all of the residential locations for all youth in the study. This meant, however, that we lost one or two years of neighborhood data for an individual youth and not the entire case. 

Census indicators for neighborhood ethnic composition (percent foreign born and African American) and socioeconomic status (social vulnerability index and occupational prestige) were derived from the Neighborhood Change Data Base ([NCDB], a Geolytics proprietary product). Using data from the NCDB, principal components analysis was used to estimate a social vulnerability index comprised of the sum of census tract percentages of poor, unemployed, renters, and female household heads in a given Census tract. Index scores range from 0 to 400 and the Cronbach’s alpha for this index was .910 (see [9] for details). An occupational prestige score was estimated based on the 1989 General Social Survey prestige score by occupation weighted by the observed proportional distribution of occupations of employees (i.e., in the labor force) within the corresponding Census tract. Occupational prestige scores ranged from 29 (when all neighborhood employees are laborers) to 62 (when all neighborhood employees are in managerial-professional occupations). For further details, see [9]. For each of these Census-derived variables, we estimated the average proportion or score when the youth were between the ages of 15 and 18.

In addition, two composite neighborhood indicators were derived from the Denver Child Study retrospective survey. Caregivers were asked to respond to a series of questions related to the assets and liabilities present in each neighborhood where their children resided during high school. These responses were then used to estimate two indices of neighborhood social capital and social problems (see [9] for details). For each year during a youth’s high school years, a social capital index (range from 0 to 6, Cronbach’s alpha = 0.747) was estimated and was incremented by “one” for the presence of each of the following assets: People who could solve neighborhood problems; would watch of our children and property; knew neighbors and their children by name; served as role models; would provide assistance when needed; and were civically engaged in the neighborhood. A social problems index (range 0–5, Cronbach’s alpha = 0.779), also was estimated for each year during high school and, was incremented by a factor of “one” with the presence of each of the following neighborhood liabilities: people selling drugs; gang activity; burglaries; robberies or muggings; and beatings or rapes. An average exposure to neighborhood social capital and problems experienced by youth between the ages of 15 and 18 was then estimated. 

Earlier models also tested the following neighborhood indicators: presence of negative peers, residential instability, presence of medical facilities, presence of other institutional resources such as parks, recreational facilities and mentoring centers, vintage of the housing stock, violent crime rates, property crime rates, and the percent of all births in the neighborhood to teens. These indicators were removed in the final analyses either because of variance inflation factors (VIFs) exceeding four suggesting multi-collinearity or they were never found predictive of teen parenting. 

We used linear interpolation or extrapolation to derive annual estimates of neighborhood conditions for the entire period between 1970 and 2007 (see detailed discussion in [9]). Since we also asked the caregiver when (if ever) the teen parenting outcome occurred, we were able to use this timing information to temporally match this outcome with corresponding neighborhood indicators. Unlike most of the prior research on teen parenting, we estimated neighborhood exposure for all time-varying predictors based on indicator averages from age 15 to: (1) Time of teen birth/fathering or, (2) for youth who did not experience this outcome, age at time of survey (for youth under 18) or (3) age 18 (for those over 18 at time of survey). 

### 3.5. Sample Characteristics

Table 1 summarizes the characteristics of the youth, caregivers and households—all of which are based on self-reported information from the primary caregivers who completed the retrospective survey. At the time of survey, the sample youth were, on average, 20.3 years old (*SD* = 4.3). Half of the sample youth were female and 54% were Latino. The average age of the caregiver when the child was born was 24.6 years. Nearly 66% of all caregivers had completed a high school diploma or higher by the time the child was age 15 and caregiver annual earnings during high school averaged $12,050. 

In the bottom panel of Table 1, we summarize the characteristics of the neighborhoods where youth resided during their high school years. Worth noting is the substantial variation around the means which shows that youth in our sample lived in a wide range of neighborhoods across the City and County of Denver. Nonetheless, DHA youth also were living in neighborhoods that were less diverse and advantaged relative to the typical youth living in Denver as a whole. The typical youth in our DHA sample resided in a neighborhood that was approximately 24% foreign born, 16% African American, had relatively low levels of occupational prestige (*M* = 37.1) suggesting an occupational mix that was laborer intensive, and experienced moderate levels of social vulnerability (*M* = 118.9) suggesting some degree of concentrated disadvantage. Study youth resided in neighborhoods during their high school years that, on average, had a social capital score of 3.7 and had approximately 1.9 neighborhood problems. The comparable averages for the City and County of Denver in 2007 were 21% foreign born, 12% African American, 41 for occupational prestige, and 96.7 for the social vulnerability score. Further, youth in Denver resided in neighborhoods that had, on average, a neighborhood social capital score of 3.3 and 1.7 neighborhood problems. 

We examined the degree to which covariates in our models were correlated with each other by estimating Pearsonian correlation coefficients (summarized in Table S1, Supplementary Materials Section 1) and variance inflation factors (VIFs) using OLS regression analyses to assess for issues of multicollinearity (results available from authors on request). Variables with VIF values above 0.5 in earlier specifications of the model were removed from the analysis due to multicollinearity and for the sake of parsimony.

The results of the analyses presented in Table S1, Supplementary Materials Section 1 suggest that we did not have significant issues of multicollinearity amongst the predictors in our final models. 

### 3.6. Analytical Approach

Our analytical approach takes DHA’s offer of a public housing unit in a particular neighborhood as an environmental “treatment” that is exogenous from the perspective of our sampled youth. Specifically, we estimate a Treatment-on-Treated (TOT) effect by utilizing instrumental variable (IV) estimates of the conditions actually experienced by youth associated with their first offered neighborhood in order to obtain an unbiased estimate of neighborhood effects on teen parenting (please note that we estimated contemporaneous and first offer models as robustness checks. We only report findings that were consistent across all three model specifications). There is a longstanding practice of using IVs as a way to overcome geographic selection bias and provide estimates of causal neighborhood effects [42,49,50,51,52]. More specifically, other researchers have employed a natural experiment analogous to ours as a source of exogenous instruments based on the neighborhoods to which subsidized housing residents were quasi-randomly assigned (e.g., [53]). In previous work with this dataset, we have thoroughly tested the same IVs used in this paper and found them to be both valid and strong [54,55,56]. 

### 3.7. Instrumentation Strategy

To address the potential of some geographic selection bias at time of initial assignment or subsequent residential moves post DHA-assignment, we estimated instrumental variables for the actual neighborhood characteristics experienced by sample youth since age 15. This instrumentation strategy employed a classic two-stage least squares procedure whereby each of the neighborhood indicators (measured as averages experienced since age 15 as explained above) was regressed first on all exogenous youth, household and caregiver variables shown in Table 1 and a set of excluded, identifying instruments (see discussion in [54]). See Table S2, Supplementary Materials Section 1 for a summary of these first stage regression results. The primary identifying instruments utilized in this process included the six neighborhood indicators associated with the dwelling unit first offered by DHA to the applicant when they reached the top of the wait list. Additionally, we included a series of dummy variables signifying the calendar year in which the initial offer was made in order to capture some of the longer-term trends in Denver neighborhoods overall, such as shifts in social vulnerability; however, calendar year was never statistically significant. Therefore, we argue that these instrument variables are valid since there is no plausible reason why the initial DHA offer of a neighborhood or the year of such offer should: (1) Influence youths’ teen parenting outcomes other than through their relationship with actual neighborhood contexts experienced; or (2) be related to unobservable caregiver characteristics related both to their neighborhood preferences and the outcome.

### 3.8. Statistical Models

In our analyses of teen parenting, we only consider births occurring after their families were quasi-randomly assigned to a DHA public housing unit, thereby preserving the value of the natural experiment as a vehicle for drawing causal inferences. We undertook a three-step analysis of the data. Our first analytical approach uses a Cox proportional hazards (PH) model to estimate the timing of teen parenting at time t for an individual *ij* with covariate vector χ:

λ(t|χ_ij_) = λ_0_(t) exp(β_1_χ_1ij_ + … + β_n_χ_nij_) = λ_0_(t) exp(χ_ij_ β)

where λ(t|χ_ij_ ) is the observed time of outcome (or the censoring time of age 18) for youth *ij* and λ_0_(t) is the baseline hazard. This modeling approach is used to predict the hazard of teen parenting using instrumental variables for the neighborhood in which caregivers’ first offered DHA unit was located (our TOT estimate). All analyses were completed in Stata, version 14. Global chi-square tests of proportionality indicate that the Cox PH models were the appropriate specifications to employ with these data.

Second, we examined the extent to which relationships observed across the full analysis sample specifications remained robust for females and males and across Latino and African American ethnic groups. 

Third, we examined the extent to which neighborhood effects were associated with the age of the youth at the time when they were first randomly assigned to their neighborhood. We generated a dummy variable indicating random assignment prior to adolescence (before age 12) and used that to create a series of interaction terms with each of the neighborhood indicators in our statistical model. The results of these analyses are discussed below and are available upon request from the authors.

## 4. Results

By the end of our study period in 2007, the teen birth rate in the City and County of Denver was 71 births per 1000 women aged 15 to 19 [57]. The percentage of all live births in Denver to teens was 13.2%. Of the 517 youth in the study sample, 96 or 18.6% birthed or fathered a child after random assignment into DHA and between the ages of 15 and 19 (see Table 2). On average, teen parents in our sample were 17.3 years old when they birthed or fathered their first child. Females were nearly three times more likely to become teen parents (27.3%) than males (9.7%) though we again note the possibility of underreporting. Latinos were slightly less likely to become teen parents (18.3%) than African American youth (18.8%).

Standardized hazard ratios and confidence intervals are presented in Table 3 for our Cox PH models predicting whether youth ever gave birth or fathered a child during their teen years. The interpretation for the hazard ratio is that a one standard deviation change in a given predictor variable is associated with the increase/decrease in the odds of birthing or fathering a child as a teen. Table 3 compares estimated parameters for the full sample of youth as well as for gender and ethnic strata for cumulative neighborhood exposure as reflected by our instrumental variables. Across all of the stratified models except for the male stratum, overall model performance was acceptable as demonstrated by the log-likelihood values and statistically significant chi-square tests. 

As shown on the left-hand side of Table 3, the results for the full analysis sample show that compared to African American males, African American females are 3.9 times more likely to become teen parents. Residing with older caregivers or ones with a high school diploma or higher, however, substantially decreases the odds of becoming a teen parent by 30% and 53%, respectively. The full sample specification also reveals two significant neighborhood predictors of teen parenting. Youth raised during adolescence in neighborhoods with one standard deviation higher percentages of foreign-born neighbors have 23% lower odds of becoming teen parents. However, residence in neighborhoods with a one standard deviation-higher level of social capital increases the odds of becoming a teen parent by nearly 29%.

Table 3 also presents the results of the stratification analyses. Overall, results were more robust for females than males; and for African American than Latino youth. African American and Latina females are 3.7 and 2.8 times more likely, respectively, to become parents during their teen years than their male counterparts. Caregiver educational attainment reduced the odds of teen parenting across all gender and ethnic strata (by 49 to 72%), and was particularly strong for African American and male youth. Caregiver age significantly reduced the odds by about a third of becoming teen parents for female and Latino youth only. The percentage of foreign-born neighbors significantly reduced the odds of becoming a teen parent for African American youth (47% reduction with a one standard deviation increase in that percentage). Additionally, social capital was a significant predictor of increased odds of teen parenting for Latino youth and marginally significant (*p* < 0.06) for female and male youth). Finally, neighborhood occupational prestige was a marginally significant predictor of decreased odds of teen parenting for African American youth. 

In analyses not presented here, we re-estimated our models using the interaction terms for age at time of initial DHA assignment and neighborhood conditions. We could find no evidence that the timing of random assignment mattered with two exceptions: the effect of social capital on teen parenting for African American youth and the effect of neighborhood problems on female youth. The risk of becoming a teen parent increased by 89% with a one standard deviation increase in social capital for African American youth; however, this effect was significantly reduced (by 32%) if they were under the age of 12 when first randomly assigned to their DHA neighborhood. In these age interaction models, we also found that one standard-deviation higher neighborhood social capital increased the odds of becoming a teen parent by 54% for females and by 62% for Latino youth.

## 5. Discussion

Our study provides a fresh contextual perspective on the factors that might lead disadvantaged youths to expeditiously transition to adulthood by becoming teen parents. We found three neighborhood contexts: nativity composition, social capital, and occupational prestige for African American youth were significant predictors of teen parenting. Each of these contexts can be understood from a collective socialization framework. There is limited evidence that relates neighborhood nativity composition [37], and social capital [58] to teen childbearing and fathering specifically. Thus, our findings in these realms expand the literature that examines how neighborhoods influence teen childbearing and fathering and how these effects differ race/ethnicity and gender. We describe each in detail below but begin with a discussion of differential effects.

### 5.1. Heterogeneity of Neighborhood Effects

Recent evidence produced for a variety of outcomes using multiple methods suggests a high degree of heterogeneity in neighborhood effects by race/ethnicity and gender [12,30,31,34,44,59,60,61]. We also found such heterogeneity for at least some neighborhood conditions. The presence of foreign-born neighbors on the risk of teen parenting produced a stronger dampening effect for African American youth. We describe the mechanisms by which we might interpret these differential results in the next section. In addition, neighborhood social capital proved to be a stronger influence on Latino youth compared to African American youth and marginally more so for males than females. Although much of the health-related neighborhood effect literature shows greater risk effects for females compared to males [34,62,63,64] there is little that explicitly looks at differential gender effects of social capital on teen parenting specifically. Nevertheless, our study adds to the mounting evidence on effect heterogeneity and suggests that future quantitative neighborhood research must probe such diversity as a matter of course or risk mis-estimating the true magnitudes of impact. Put differently, the strategy should not be to test whether there is a neighborhood effect but rather to investigate for whom, when and where there is an effect [30,31]. 

### 5.2. Neighborhood Nativity Composition

In our full sample, we found the immigrant composition of the neighborhood’s population to have an important, inverse relationship with teen parenting; this relationship was particularly strong for African American youth. These results are consistent with the notion that immigrant culture, which in the Denver context is primarily Latino (especially Mexican heritage), plays a powerful normative, role-modeling, and behavioral monitoring function that discourages early childbearing, especially outside of marriage. 

Our findings extend the literature on neighborhood ethnic and nativity composition and teen parenting. Brewster, Billy and Grady [65], found that higher proportions of Latinos in the neighborhood were related to a decreased hazard of first intercourse, and Browning, Burrington, Levanthal and Brooks-Gunn [7], found that in neighborhoods with high levels of immigrant and Latino concentration, the likelihood of having had no sexual partner as a teen increased exponentially. Although each of these studies is distinct from ours in a number of ways, they share one important conclusion: Concentrations of immigrant and/or Latino populations within neighborhoods can be a significant protective factor for the youths who reside in these neighborhoods. The protective quality may be due in large part to the unique strengths, social cohesion, and informal social control that may be present in immigrant enclaves [66,67]. Moreover, immigrant Latino cultural values (e.g., traditional values related to sexual behavior, tighter surveillance of adolescent females, and discouragement of non-marital family formation see [7,68]) also may protect against early childbearing and fathering outside the enclave, as suggested by the strong effects on African American youth. During the period of our study, immigrants moved into Denver neighborhoods, particularly in Northeast Denver, that had been occupied previously by large fractions of African Americans. In addition to the potential for increased adult monitoring of all neighborhood youth, African American youth may also be exposed to other adult role models and two-parent or multi-generational families that foster norms around family formation that effectively delay the transition to parenthood for African American youth. Alternatively, Browning and colleagues [7] discuss how ethnic and racial heterogeneity within neighborhoods might limit communication and interaction among residents and effectively hinder the development of network ties across racial/ethnic groups. This theory of ethnic heterogeneity provides an alternative explanation as to the protective nature of foreign-born composition for African American youth in Denver. If racial tension exists between African Americans and Latinos in Denver, it would follow a risk-model of ethnic heterogeneity that African American youth in concentrated immigrant (and in this case, Latino) neighborhoods would experience social isolation. This social isolation may actually lead to decreased opportunities for engaging in risky sexual behaviors with one’s peers [12]. 

### 5.3. Neighborhood Social Capital

Our most unexpected finding was associated with the links between neighborhood social capital and teen parenting. Contrary to earlier studies that suggest higher levels of social capital decrease the odds of teen parenting [17,69], we found that greater social capital in the teen’s neighborhood increased the likelihood of teen parenting, particularly for Latino youth. Part of the difference in findings may be attributable to geographic scale. While some studies that show a negative relationship between social capital and teen parenting have used larger geographic scales such as the state or region [17,69], our indicator for social capital is for the more immediate neighborhood. If social capital is viewed particularly through the lens of more localized network ties and trust (bonding social capital), these findings may not be so counterintuitive. One could also argue that ties to local networks of youth and adults who offer emotional as well as instrumental support to teen mothers and fathers afford neighborhood teens with the ability to select early parenthood as a viable option in the transition to adulthood. The existence and utilization of these additional sources of informal support reduce the deleterious consequences to teens and their children. Further, this option may be sustained by local community norms that value early parenthood for both adolescent girls and boys. Finally, if neighborhood teens trust these community members, they are more likely to adopt the same normative values and beliefs (e.g., social contagion per Crane, [1]). 

Yet, recent work by Siebold-Simpson and Morrison-Beedy [70] suggests that the pathway linking social capital with teen parenting is more complex, pulling youth in multiple directions. When the teen’s social networks are expanded to include individuals residing outside of the more localized neighborhood (bridging social capital), they found that the presence of these expanded networks decreased youth’s positive attitudes toward early parenting and reduced the risk of teen childbearing. They argue that expanding the horizons of low-income, minority youth to include others from different areas and backgrounds fosters positive attitudes toward alternative paths to adulthood and deferring early parenting. In a similar vein, a recent qualitative study found that Latino youths described families and immediate surroundings rich with bonding social capital, but despite these youth’s strong social ties, they often lacked the bridging social capital that would help youth navigate complex structures and systems (e.g., educational settings and college applications) that would, in the long-term promote delayed childbearing/fathering and overall upward social mobility [71]. Ultimately, our study’s findings suggest that future inquiry should attempt to measure each domain of social capital and consider neighborhood scale and respondents’ operationalization of such in order to further sort out the complexity of this finding.

### 5.4. Neighborhood Socioeconomic Status and Teen Parenting

With one exception, we did not find significant relationships between traditional, census-based indicators of neighborhood social vulnerability or affluence and teen parenting. Although occupational prestige was associated with decreased odds of teen parenting among African American youth, this was not the case with any other strata. Further, our failure to find a relationship between social vulnerability and teen parenting is quite different from much of the prior literature on the topic suggesting that previous studies that lacked the ability to control for other neighborhood contexts and/or did not account for parental selection bias in their empirical methods may have produced incomplete inferences. 

## 6. Limitations

Despite the merits of our study, we acknowledge a number of limitations. First, our study looks only at neighborhood exposure during high school years; observed neighborhood effects might be different if they were quantified as cumulative effects of neighborhood exposure throughout childhood. Second, we recognize the potential shortcomings of using caregiver reports of teen parenting, including recall error. Although caregivers would most likely be aware of whether their daughter gave birth as a teen, it is possible that they could be less aware of whether their sons had fathered children. In this case, we may have underestimated the prevalence of teen fathering in our sample and introduced measurement error. Third, although we have examined a more comprehensive set of neighborhood indicators than is typical, we recognize their limitations, especially in describing the physical/ecological environs nearby the home and quality of local public and institutional resources (e.g., features of the school youths attend). Fourth, the study’s findings can only be generalized to Latino and African American youth. Finally, evidence from Denver likely understates the consequences of highly vulnerable neighborhood environments on teen parenting. Denver has relatively few areas of concentrated neighborhood disadvantage for either Latinos or African Americans. In addition, Denver’s unified city–county governmental and social service delivery systems produce less variation in access to resources than found in many other metropolitan areas in the United States. 

## 7. Conclusions

Data from a natural experiment in Denver have allowed us to undertake a more nuanced analysis of the causal impact of neighborhood environments on teen childbearing and fathering. We found that social capital, nativity composition and neighborhood affluence were important predictors of teen childbearing and fathering, though in heterogeneous and sometimes unexpected ways. Our study highlights the role that neighborhood context plays in shaping the behaviors and ultimately the trajectories of low-income, minority youth. Specifically, our study found that, at least in part, neighborhood effects on teen parenting operate differentially based on race and gender—adding to the growing body of evidence regarding differential exposure and differential effects across groups. Our results suggest that certain aspects of neighborhood could be leveraged to reduce the incidence of teen childbearing and fathering. With progressive policy changes in public housing, Low-Income Housing Tax Credit and Housing Choice Voucher programs that expand options for low-income, minority teens to access immigrant-rich and higher-status neighborhoods, we might see a reduction in teen parenting and thus an expansion of opportunities for at-risk youths.

## Figures and Tables

**Table 1:** Descriptive Characteristics of Youth, Caregivers, Households and Neighborhoods for Analysis Sample (N = 517).

Variables	M or %	SD or *n*	Min	Max	*n* of Items	α
*Youth Characteristics*						
Age at time of survey	20.3	4.3	15	35		
Age when first random assignment to DHA occurred	7.9	4.6	1	17		
Gender and ethnicity of youth						
Latina female	25.1%	130				
Latino male	28.6%	148				
African American female	25.1%	130				
African American male	21.1%	109				
*Caregiver and Household Characteristics (unless specified otherwise, values are averages during high school)*						
Caregiver age at time of child’s teen birth	24.6	6.7	11.4	62.9		
Caregiver immigrant status						
U.S. born	87.0%	450				
Immigrant	13.0%	67				
Caregiver educational attainment						
No high school diploma	34.2%	177				
High school diploma or higher	65.8%	340				
Caregiver earnings (in dollars)	$12,050	$12,991	$1	$66,353		
Natural log of caregiver earnings (in dollars)	6.30	4.55	0.10	11.10		
*Neighborhood Characteristics (values are averages during high school)*						
Social vulnerability score (range 0–400)	119.0	50.9	17.9	289.0	4	0.91
Occupational prestige score (range 29–62)	37.1	3.2	31.4	47.3		
Percent African American residents	16.3	19.0	0.2	99.5		
Percent foreign born residents	23.8	12.1	0.3	53.0		
Social capital score (range 0–6)	3.7	1.5	0.0	6.0	6	0.75
Neighborhood problems score (range 0–6)	1.9	1.8	0.0	6.0	6	0.78

**Table 2:** Prevalence and Age of Teen Childbearing/Fathering ^a^ for Denver Child Study Samples and Denver, Colorado, 2007 ^b^.

Ever Birthed or Fathered Child between 15 and 19	M	*SD*	Min	Max
Full sample (N = 517)	0.186	0.389	0	1
Males (N = 257)	0.097	0.297	0	1
Females (N = 260)	0.273	0.446	0	1
Latinos (N = 278)	0.183	0.388	0	1
African Americans (N = 239)	0.188	0.392	0	1
Percent of live births occurring to teens aged 15 to 19				
City and County of Denver, 2007	0.132	0.068	0.032	0.429

Notes: ^a^ Prevalence of teen childbearing/fathering based on author estimates using data derived from caregiver retrospective reports of these behaviors for children aged 12 and older at the time of the *Denver Child Study* survey; ^b^ Prevalence of teen childbearing for the City and County of Denver based on estimates derived by the authors from data in the *Neighborhood Facts Database*. The percentage of live births to teens declined from 15.2 in 1987 to 13.5 in 2007.

**Table 3:** Standardized Cox PH Models Predicting Hazards of Giving Birth or Fathering a Child between ages 15 and 19 Years, Instrumental Variable Models.

	Full Sample			Females			Males			Latino			African American		
	HR	95% CI		HR	95% CI		HR	95% CI		HR	95% CI		HR	95% CI	
*Child Characteristics*															
Latina female (omitted = African American male)	2.894 +	0.981	8.532												
Latino male	1.094	0.384	3.117												
African American female	3.884 **	1.802	8.371												
African American (omitted = no)				1.347	0.519	3.490	0.927	0.178	4.831						
Female (omitted = no)										2.837 ***	1.651	4.875	3.721 **	1.640	8.444
*Caregiver and Household Characteristics (unless otherwise noted, values reflect averages during high school)*															
Caregiver age at time of child’s birth	0.692 **	0.531	0.901	0.631 **	0.462	0.863	0.832	0.551	1.257	0.678*	0.499	0.920	0.812	0.522	1.261
Caregiver educational attainment (omitted = no degree)															
H.S. diploma or higher	0.475 **	0.286	0.791	0.511 *	0.297	0.877	0.281 *	0.095	0.832	0.522 *	0.287	0.951	0.331 ***	0.171	0.640
Natural log of caregiver earnings (in dollars)	1.009	0.811	1.254	0.991	0.789	1.245	1.105	0.725	1.684	0.916	0.686	1.224	1.225	0.795	1.888
*Neighborhood Characteristics (values reflect averages during high school)*															
Social vulnerability score (range 0–400)	0.979	0.715	1.340	1.131	0.831	1.539	0.581	0.298	1.133	0.949	0.604	1.490	0.842	0.559	1.270
Occupational prestige score (range 29–62)	0.865	0.647	1.157	0.969	0.736	1.276	0.577	0.285	1.170	1.038	0.766	1.407	0.666 +	0.439	1.011
Percentage African American residents	1.015	0.693	1.488	1.026	0.669	1.572	1.071	0.522	2.198	1.355	0.697	2.632	0.773	0.480	1.245
Percentage foreign born residents	0.762 *	0.578	1.007	0.830	0.617	1.117	0.598	0.342	1.048	0.905	0.650	1.259	0.535 **	0.361	0.792
Social capital score (range 1–6)	1.286 *	1.030	1.605	1.271 +	0.994	1.626	1.516 +	0.978	2.350	1.426 **	1.021	1.992	1.121	0.828	1.519
Neighborhood problems score (range 1–6)	0.910	0.717	1.156	0.790	0.602	1.036	1.419	0.916	2.199	0.885	0.654	1.198	0.933	0.644	1.353
Number of observations	517			260			257			278			239		
Number of clusters	283			185			191			157			130		
Number of failures	96			71			25			51			45		
Time at risk	8795			4458			4461			4796			4123		
Log-Likelihood	−540.692			−355.91			−125.925			−255.243			−215.596		
Chi-square	70.79 ***			25.66 **			13.02			44.08 ***			46.17 ***		

Notes: HR = Hazard ratio (exponentiated coefficients); 95% confidence intervals are presented in the second and third columns for each model specification; models are adjusted for clustering of siblings within families by utilizing robust standard errors; time at risk is measured using child years experienced prior to the occurrence of becoming a teen parent or through age 18 if the youth did not parent a child prior to age 19. * *p* < 0.05; ** *p* < 0.01; *** *p* < 0.001; + indicates marginal significance (*p* < 0.06).

## Supplementary Materials

The following are available online at www.mdpi.com/2227-9032/6/1/7/s1. Section 1: Supplementary Analyses: Correlations and First Stage Regression Results.

Supplementary File 1
