# Supplementary material for: How Neighborhood Effects Vary: Childbearing and Fathering among Latino and African American Adolescents
Source: Healthcare (Basel). 2018 Jan 18;6(1):7. doi: 10.3390/healthcare6010007 (PMC5872214; doi:10.3390/healthcare6010007)
Supplement: Supplementary File 1 [file healthcare-06-00007-s001.pdf]

Supplementary Materials: Neighborhood Effects on Childbearing and Fathering among Latino and African American Adolescents

Jessica L. Lucero, Anna Maria Santiago and George C. Galster

1. Supplementary Analyses – Correlations and First Stage Regression Results

Table S1. Child, Caregiver, Household and Neighborhood Characteristics: Correlations (N=517)

Variables	1	2	3	4	5	6	7	8	9	10	11
1. Youth gender[a]	-										
2. Youth ethnicity[b]	0.076	-									
3. Caregiver age at time of child's teen birth	-0.082*	-0.105	-								
4. Caregiver educational attainment when child was 15[c]	0.008	0.277*	-0.032	-							
5. Average annual caregiver income during high school (in dollars)	0.04	0.209*	-0.145*	0.226*	-						
6. Social vulnerability score (range 0-400)	0.006	-0.001	0.342*	-0.064	-0.105	-					
7. Occupational prestige score (range 29-62)	-0.197*	0.275*	-0.152*	0.016	0.127*	-0.450*	-				
8. Percentage African American residents	0.052	0.806*	-0.027	0.239*	0.156*	0.138*	0.122*	-			
9. Percentage foreign born residents	0.08	-0.124*	-0.322*	-0.089*	-0.059	0.068	-0.412*	-0.130*	-		
10. Social capital (range 0-6)	-0.081	-0.095	0.172*	0.04	0.002	-0.086	-0.061	-0.167*	-0.013	-	
11. Neighborhood problems (range 0-6)	0.108*	0.279*	-0.132*	0.146*	-0.044	0.190*	0.021	0.405*	0.127*	-0.105*	-

[a] Youth gender: 0=male, 1=female. [B] Youth ethnicity: 0=Latino, 1=African American. [C] Caregiver educational attainment when child was 15: 0=no degree, 1: H.S. diploma or higher.

*p<.05.

Table S2. First Stage Regression Results for Neighborhood Conditions at Time of Teen Childbearing or Fathering

	Dependent Variables: Neighborhood Conditions at Time of Teen Childbearing or Fathering											
	Social Vulnerability		Percent African American		Percent Foreign Born		Occupational Prestige		Social Capital		Neighborhood Problems	
Exogenous Predictors	b	SE	b	SE	b	SE	b	SE	b	SE	b	SE
Covariates at Time of First Offer												
Female (omitted=male)	3.164	3.963	-0.002	1.329	0.750	0.861	-0.693**	0.243	-0.016	0.120	0.167	0.129
Ethnicity omitted=Latino)	-5.580	5.746	13.827***	2.234	-4.116*	1.415	0.888*	0.426	0.033	0.188	0.043	0.215
Caregiver age	1.377**	0.491	0.122	0.102	-0.351***	0.075	-0.0523*	0.024	0.023*	0.012	-0.010	0.012
Caregiver has high school diploma or higher	-7.871	5.645	-0.134	2.253	-1.562	1.346	-0.071	0.364	0.104	0.190	0.031	0.207
Natural log of household income	-0.184	0.601	-0.062	0.204	0.133	0.135	0.005	0.037	0.010	0.020	-0.012	0.022
Timing of DHA First Offer (omitted=pre1990)												
offer1990-91	0.688	8.405	-3.964	3.237	1.201	2.006	0.408	0.529	-0.231	0.271	-0.071	0.273
offer1992-93	-13.406	9.236	-1.987	3.765	1.435	3.184	0.663	0.682	-0.120	0.336	0.479	0.465
offer1994-95	4.333	10.755	-4.427	3.447	2.362	2.247	-0.062	0.699	0.222	0.313	0.665	0.444
offer1996-97	11.786	13.304	-4.944	3.652	1.971	2.541	-0.712	0.681	-0.180	0.337	0.685	0.423
offer1998-99	-4.913	13.952	-8.902	3.559	6.841	3.158	-0.403	0.732	-0.426	0.438	-0.205	0.409
offer2000-01	-0.144	12.850	-7.004	4.273	4.152	3.152	-0.455	0.833	-0.456	0.423	0.688	0.488
offer2002-03	19.716	19.244	-6.240	4.177	0.502	3.533	-0.601	0.917	-0.717	0.444	0.946	0.524
offer2004-05	-2.967	-21.581	-10.174	6.667	4.048	4.030	1.165	1.253	-0.288	0.613	0.784	0.971
Neighborhood Conditions at Time of First Offer												
Social vulnerability score (range 0-100)	0.441***	0.062	0.027	0.021	0.008	0.014	-0.001	0.003	-0.001	0.002	0.003	0.002
Percentage African American residents	10.428	12.674	37.462***	6.682	14.076***	3.143	-0.304	0.916	-0.660	0.525	1.515	0.614
Percentage foreign born residents	45.005	53.160	9.464	14.659	71.589***	11.643	-1.286	2.956	0.367	1.537	1.164	1.636
Occupational prestige score (range 0-100)	0.885	1.595	0.107	0.500	-0.474	0.350	0.516***	0.100	-0.040	0.039	-0.008	0.047
Social Capital	-0.650	1.526	-0.272	0.464	0.260	0.365	0.066	0.093	0.313***	0.054	-0.054	0.055

Neighborhood Problems	-1.707	1.549	0.430	0.510	-0.099	0.351	0.203*	0.098	0.068	0.055	0.375***	0.060
Constant	-28.434	60.449	-5.823	19.865	20.376***	-3.652	20.646***	3.965	3.451*	1.637	0.717	1.992
Observations	550		542		1024		542		569		569	
R^2	0.278		0.403		0.215		0.282		0.192		0.235	
F-test	5.43***		10.54***		13.63***		8.12***		3.46***		5.01***	

SOURCE. – Authors' tabulations.

NOTE. – Standard errors in Column 2 are for each dependent variable.

* p<.05. ** p <.01. *** p <.001.
